# Application of cell-derived exosomes in the hematological malignancies therapy

**DOI:** 10.3389/fphar.2023.1263834

**Published:** 2023-09-08

**Authors:** Kazem Ghaffari, Amin Moradi-Hasanabad, Ali Sobhani-Nasab, Javad Javaheri, Ali Ghasemi

**Affiliations:** ^1^ Department of Basic and Laboratory Sciences, Khomein University of Medical Sciences, Khomein, Iran; ^2^ Autoimmune Diseases Research Center, Shahid Beheshti Hospital, Kashan University of Medical Sciences, Kashan, Iran; ^3^ Physiology Research Center, Institute for Basic Sciences, Kashan University of Medical Sciences, Kashan, Iran; ^4^ Department of Health and Community Medicine, School of Medicine, Arak University of Medical Sciences, Arak, Iran; ^5^ Department of Biochemistry and Hematology, Faculty of Medicine, Semnan University of Medical Sciences, Semnan, Iran; ^6^ Cancer Research Center, Semnan University of Medical Sciences, Semnan, Iran

**Keywords:** exosomes, hematological, therapeutic agents, cancer, biogenesis

## Abstract

Exosomes are small membrane vesicles of endocytic origin that are produced by both tumor and normal cells and can be found in physiological fluids like plasma and cell culture supernatants. They include cytokines, growth factors, proteins, lipids, RNAs, and metabolites and are important intercellular communication controllers in several disorders. According to a vast amount of research, exosomes could support or inhibit tumor start and diffusion in a variety of solid and hematological malignancies by paracrine signaling. Exosomes are crucial therapeutic agents for a variety of illnesses, such as cancer and autoimmune diseases. This review discusses the most current and encouraging findings from *in vitro* and experimental *in vivo* research, as well as the scant number of ongoing clinical trials, with a focus on the impact of exosomes in the treatment of malignancies. Exosomes have great promise as carriers of medications, antagonists, genes, and other therapeutic materials that can be incorporated into their core in a variety of ways. Exosomes can also alter the metabolism of cancer cells, alter the activity of immunologic effectors, and alter non-coding RNAs, all of which can alter the tumor microenvironment and turn it from a pro-tumor to an anti-tumor milieu. This subject is covered in the current review, which also looks at how exosomes contribute to the onset and progression of hematological malignancies, as well as their importance in diagnosing and treating these conditions.

## Introduction

Hematological malignancies pose significant challenges in terms of treatment options and patient outcomes (Mohseni Afshar et al., 2022). Current therapeutic approaches often have limitations, including systemic toxicity and the inability to specifically target malignant cells (Belfiore et al., 2018). The application of cell-derived exosomes in hematological malignancies therapy holds great promise and significance. Exosomes, as natural carriers of bioactive molecules, offer several advantages, such as targeted delivery, reduced off-target effects, and potential immunomodulatory effects (Dimik et al., 2023). By utilizing exosomes as therapeutic vehicles, it may be possible to enhance the efficacy of drug delivery, overcome treatment resistance, and modulate the tumor microenvironment in hematological malignancies (Xia et al., 2020). Therefore, understanding the potential of exosomes in this context is of immense importance, as it may contribute to the development of novel and more effective treatment strategies with improved outcomes for patients with hematological malignancies.

Exosomes are small extracellular vesicles that play a crucial role in intercellular communication and have garnered significant attention in the fields of tumor biology and therapeutics (Yokoi and Ochiya, 2021). These nanosized vesicles are secreted by various cell types and are enriched with proteins, nucleic acids, and lipids, reflecting the molecular composition of their parent cells. Their ability to transport and deliver cargo molecules, including proteins, RNA, and miRNAs, to recipient cells has highlighted their importance in cellular signaling and disease progression (Veerman et al., 2019; Tenchov et al., 2022).

In recent years, exosomes have emerged as key mediators in the tumor microenvironment, facilitating the transfer of bioactive molecules between cancer cells and nearby or distant cells. Through their cargo, exosomes can modulate cellular processes such as proliferation, angiogenesis, immune response, and metastasis, thereby influencing tumor development and progression (Jia et al., 2017; Jena and Mandal, 2021).

The unique characteristics of exosomes, including their stability in various body fluids, protection against degradation, and ability to cross biological barriers, make them attractive candidates for therapeutic applications (Alzhrani et al., 2021). Researchers have explored the potential of exosomes as natural drug delivery vehicles, harnessing their ability to target specific cells or tissues. By engineering exosomes or loading them with therapeutic agents, it is possible to enhance drug efficacy, reduce off-target effects, and overcome drug resistance (Kibria et al., 2018; He et al., 2022). Moreover, exosomes have shown promise as diagnostic and prognostic biomarkers for various diseases, including cancer. Their presence in bodily fluids, such as blood, urine, and saliva, allows for non-invasive and readily accessible sampling, making them valuable tools for disease detection, monitoring, and personalized medicine (Bei et al., 2017; Huang and Deng, 2019).

More details on the significance of exosomes in intercellular communication, tumor biology, and potential therapeutic applications are given below.

1. Intercellular communication: exosomes are involved in a wide range of cellular communication processes. They can transfer various biomolecules, including proteins, lipids, and nucleic acids, between cells. These cargo molecules can exert functional effects on recipient cells by altering gene expression, signaling pathways, and cellular behavior. Exosomes have been found to participate in intercellular communication in diverse physiological contexts, such as immune response modulation, neuronal signaling, and tissue repair (Ludwig and Giebel, 2012; Ahmadi and Rezaie, 2020; Huo et al., 2021).
2. Tumor biology: in the context of cancer, exosomes have emerged as key players in tumor progression and metastasis. Tumor-derived exosomes can promote tumor growth by enhancing angiogenesis, and the formation of new blood vessels that supply nutrients to the tumor. They can also suppress the immune system’s response to cancer cells, allowing tumors to evade immune surveillance. Moreover, exosomes can prepare distant sites for tumor metastasis by facilitating the recruitment and reprogramming of cells in pre-metastatic niches. These processes contribute to the aggressiveness and spread of cancer (Aslan et al., 2019; Li et al., 2019; Zhou et al., 2021).
3. Therapeutic applications: the unique properties of exosomes make them attractive for therapeutic applications. One promising approach is to use exosomes as natural carriers for delivering therapeutic molecules, such as drugs or nucleic acids, to target cells. Exosomes can be engineered to express specific surface proteins or loaded with therapeutic cargo, enabling targeted delivery to diseased tissues while minimizing off-target effects. Furthermore, exosomes have inherent stability and biocompatibility, making them suitable for drug delivery systems. Research is ongoing to optimize exosome-based therapies and improve their efficacy in treating various diseases, including cancer, neurodegenerative disorders, and cardiovascular diseases (Sun et al., 2020; Ke and Afonin, 2021; Bashyal et al., 2022).
4. Diagnostic and prognostic biomarkers: exosomes have garnered significant interest as potential biomarkers for disease diagnosis and prognosis. Their presence in various bodily fluids, including blood, urine, and cerebrospinal fluid, allows for non-invasive sample collection. The molecular cargo within exosomes reflects the physiological and pathological states of their parent cells, making them valuable for disease detection and monitoring. Analyzing the content of exosomes, such as specific proteins, RNA molecules, or DNA mutations, can provide insights into disease subtypes, treatment response, and disease progression. This information can aid in personalized medicine approaches, guiding treatment decisions, and monitoring patient outcomes (Li et al., 2015; Alipoor et al., 2018; Yu et al., 2022).
5. Future directions: the field of exosome research is still evolving, and there are several exciting areas for future exploration. Researchers are actively investigating the mechanisms of exosome biogenesis, cargo sorting, and release, as well as the specific signaling pathways involved in exosome-mediated communication (Zhang et al., 2019). Additionally, efforts are being made to standardize exosome isolation and characterization methods to ensure reliable and reproducible results. Further advancements in exosome engineering, cargo loading techniques, and targeted delivery strategies will contribute to the development of innovative therapeutic interventions.

In overall, exosomes have far-reaching implications in intercellular communication, tumor biology, and therapeutic applications. Their ability to transfer signaling molecules, influence cellular behavior, and serve as diagnostic biomarkers make them a fascinating area of research with broad implications for various fields of medicine. Continued investigations into exosome biology and engineering hold great promise for advancing our understanding and harnessing the potential of these remarkable nanovesicles.

Exosomes have emerged as an attractive area of research due to several gaps in knowledge and limitations in existing treatments in various fields of medicine. These include.

1. Limited drug delivery systems: current drug delivery systems often face challenges such as poor bioavailability, off-target effects, and limited ability to cross biological barriers (Adepu and Ramakrishna, 2021). Exosomes, with their inherent ability to transport cargo molecules, including therapeutic agents, offer a potential solution to overcome these limitations (O'Loughlin et al., 2012). By engineering exosomes or loading them with specific therapeutic agents, it is possible to enhance drug delivery to target cells or tissues, thereby improving treatment efficacy and reducing side effects (Liang et al., 2021).
2. Incomplete understanding of intercellular communication: the complex mechanisms of intercellular communication, particularly in diseases like cancer, are not fully understood. Exosomes play a crucial role in this communication process by transferring bioactive molecules between cells. Investigating the cargo and signaling pathways within exosomes can provide valuable insights into disease progression, resistance mechanisms, and potential therapeutic targets (Xie et al., 2017).
3. Diagnostic challenges: traditional diagnostic methods often require invasive procedures or may lack sensitivity and specificity. Exosomes, being present in various bodily fluids, offer a non-invasive and readily accessible source of biomarkers (Zhou et al., 2020). However, further research is needed to identify specific exosomal biomarkers and develop standardized diagnostic assays to improve disease detection, monitoring, and personalized medicine approaches.
4. Limited treatment options for neurodegenerative diseases: neurodegenerative diseases, such as Alzheimer’s and Parkinson’s, currently lack effective treatments that can halt or reverse disease progression (Neef et al., 2011). The unique properties of exosomes, including their ability to cross the blood-brain barrier and transport therapeutic molecules, make them a promising avenue for developing novel treatments for these diseases (Li et al., 2021). However, extensive research is still required to understand the intricacies of exosome-mediated delivery and to optimize therapeutic strategies.
5. Tumor microenvironment complexity: tumors are highly heterogeneous and consist of cells with distinct characteristics (Marusyk et al., 2012). The tumor microenvironment, which includes interactions between cancer cells, immune cells, and stromal cells, plays a crucial role in tumor development and response to treatment (Anderson and Simon, 2020). Exosomes act as mediators in this complex microenvironment, facilitating communication and modulating cellular processes (Maia et al., 2018). Understanding the specific roles of exosomes in tumor progression and therapy resistance can provide new insights and potential targets for more effective anticancer strategies.

Overall, exosome research addresses the current gaps in knowledge and limitations in existing treatments by offering a promising platform for drug delivery, understanding intercellular communication, improving diagnostics, exploring neurodegenerative disease treatments, and deciphering the complexities of the tumor microenvironment. Continued research in these areas will contribute to advancements in medicine and potentially lead to improved patient outcomes.

Due to this issue, this study aims to explore and evaluate the potential application of cell-derived exosomes in the therapy of hematological malignancies, such as leukemia, lymphoma, and multiple myeloma. The aim is to investigate the unique properties of exosomes, including their ability to deliver therapeutic agents and modulate immune responses, and assess their effectiveness in targeted drug delivery and immunomodulation within the context of hematological malignancies. By critically analyzing the existing literature and synthesizing the findings, this study aims to provide a comprehensive academic overview of the current state of research, identify gaps in knowledge, and propose future directions for the development and optimization of exosome-based therapies in hematological malignancies.

## Biogenesis of exosome

Exosome biogenesis is a complex process involving multiple steps within the cell. This process includes the following steps.

1. Formation of early endosomes: the biogenesis of exosomes begins with the formation of early endosomes, which are membrane-bound compartments derived from the plasma membrane (Mincheva‐Nilsson and Baranov, 2014). This process can be triggered by various stimuli, such as cellular activation, stress, or signaling pathways. The early endosomes internalize extracellular materials, including proteins, lipids, and nucleic acids, through processes like endocytosis and phagocytosis (Abels et al., 2016; Gurung et al., 2021).
2. Maturation into late endosomes: early endosomes mature into late endosomes through a process called maturation or maturation transition (van Weering et al., 2012). This maturation involves changes in the endosomal membrane composition and the inward budding of intraluminal vesicles (ILVs) within the late endosomal compartment (Nour and Modis, 2014; Van Niel et al., 2018). ILVs are formed by the invagination of the endosomal membrane, resulting in the encapsulation of specific cargoes within vesicles (Xu et al., 2018).
3. Formation of Multivesicular Bodies (MVBs): late endosomes containing ILVs are referred to as MVBs. MVBs can further undergo two distinct pathways: the recycling pathway or the pathway leading to exosome release (Rainero and Norman, 2013; Raudenska et al., 2021). In the recycling pathway, MVBs can fuse with the plasma membrane, releasing ILVs back into the extracellular space as exosomes (Van Niel et al., 2006; Cocucci and Meldolesi, 2015). In contrast, the pathway leading to exosome release involves the fusion of MVBs with lysosomes, resulting in the degradation of their content (Buschow et al., 2005; Villarroya-Beltri et al., 2016).
4. Exosome release: when MVBs fuse with the plasma membrane, the ILVs within them are released into the extracellular space as exosomes (Janas et al., 2015). This process of exosome release allows the transfer of bioactive molecules, including proteins, lipids, and nucleic acids, from the originating cell to recipient cells (Wang W. et al., 2020). Exosomes can then travel through body fluids, such as blood or cerebrospinal fluid, to reach target cells in local or distant locations (Zhang and Grizzle, 2014; Bátiz et al., 2016).

## Types of exosome

Different types of extracellular vesicles (EVs), including microvesicles (MVs), apoptotic bodies, and exosomes, have been categorized based on their origin, biogenesis, and functions (Minciacchi et al., 2015). The term “exosome” specifically refers to a group of nanovesicles with a diameter of 30–150 nm. These nanovesicles are generated within endosomes and released into the extracellular environment (Belfiore et al., 2018). The biogenesis of exosomes involves three distinct endosomal processes: the formation of endocytic vesicles, the generation of multivesicular bodies (MVBs), and the release of exosomes (Abels et al., 2016; Boriachek et al., 2018). Another mechanism to cease intracellular biogenesis is the degradation of exosomes in lysosomes. During the initial stage of exosome biogenesis, a portion of the cell membrane invaginates and forms an early endosome. Subsequently, early endosomes mature into late endosomes, which contain intraluminal vesicles (ILVs) such as MVBs. The formation of ILVs is regulated by endocytosis-associated proteins, the lipid raft complex, and the Rab and Ras GTPase families (Abels et al., 2016; Kim et al., 2018). MVBs serve as precursors for exosomes, as the composition of exosomes is derived from ILVs. Thus, exosome biogenesis is closely linked to endosomal maturation (Huotari and Helenius, 2011; Kim et al., 2018). Eventually, ILVs are released from MVBs, giving rise to exosomes in the extracellular milieu. The production of exosomes can occur independently or rely on the sorting machinery of the endosomal complex, which is essential for the transportation of proteins (Huotari and Helenius, 2011; Kim et al., 2018).

Exosomes are classified as either artificial or natural exosomes depending on whether they have undergone artificial modification. Animal- and plant-derived exosomes can be found in natural exosomes. Animal-derived exosomes can be classified into normal and tumoral exosomes since exosomes are produced in both normal and tumorous conditions (Zhang Y. et al., 2020; Song et al., 2021).

**FIGURE 1:**
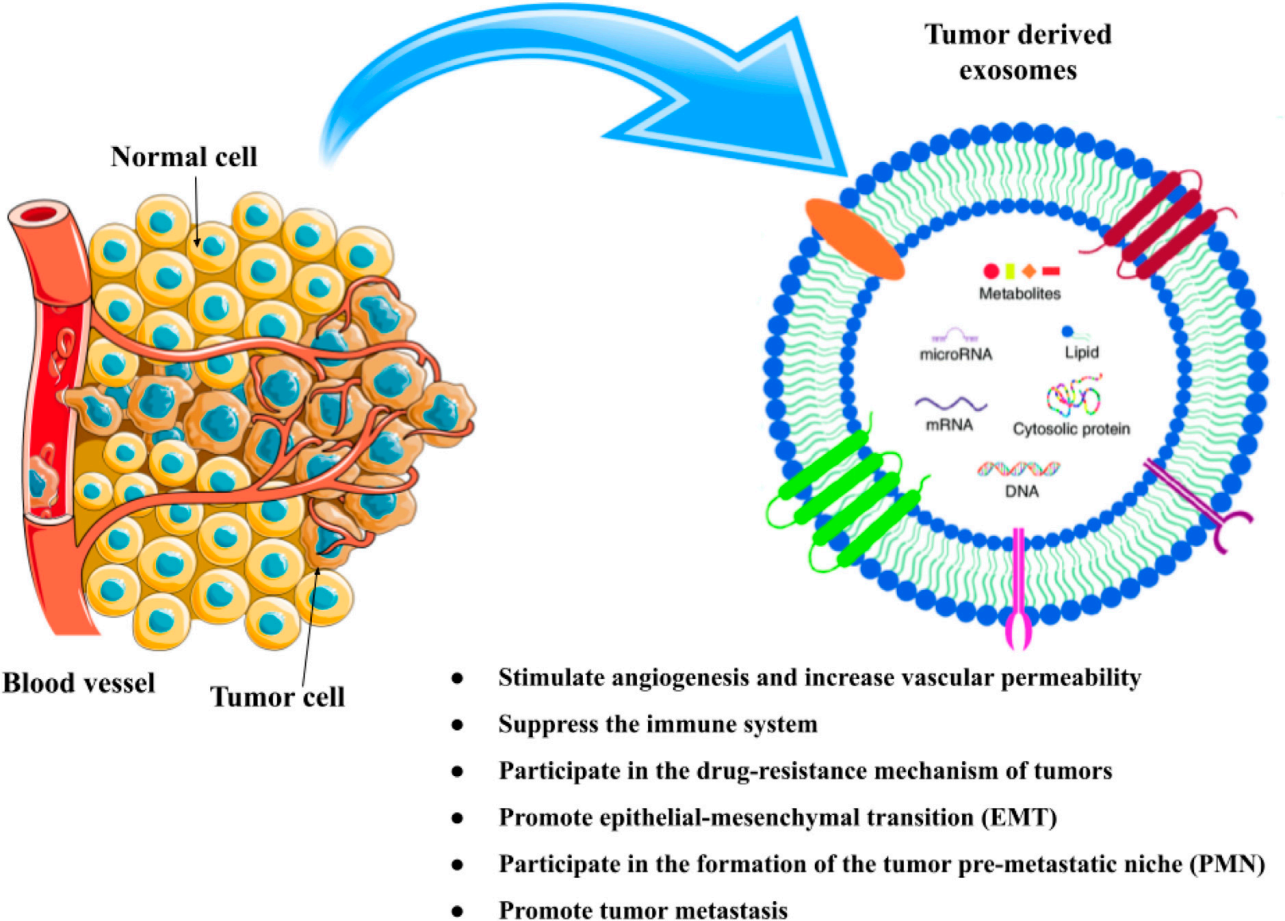
The composition and function of exosomes in cancer. Different kinds of proteins, nucleic acids, lipids, and metabolites can be found in exosomes.

Exosomes are released by various cell types and have an immune-modulating role (Song et al., 2021). The majority of healthy cells, including B-cell, T cells, human umbilical cord, macrophages, natural killer (NK) cells, vein endothelial cells, dendritic cells (DC), and mesenchymal stem cells (MSC), can produce exosomes (Wang J-H. et al., 2020). They are composed of lipid bilayers and are identified by their hallmarks such as different types of lipids including cholesterol, ceramide, phosphatidylserine, and sphingomyelin, protein families such as β-galactosidase, glycoproteins N-linked glycans and O-linked glycans, signaling receptors such as FasL, TNF receptor and TfR, adhesion molecules such as P-selectin, α and β-integrin, cytoskeletal proteins include actin, cofilin and tubulin, growth factors and cytokines such as TGF-β, TNF-α, and TRAIL), ESCRT machinery such as TSG101 and ALIX), tetraspanins include CD82, CD81, CD63 and CD9, heat shock proteins (HSP) such as HSP-60, 70, and 80)), and nucleic acids includes DNA, miRNA, non-coding RNA and mRNA (Wang J-H. et al., 2020; Gurung et al., 2021). They play a crucial part in intercellular signaling, which is engaged in a variety of physiologic and pathological processes. Additionally, adipocytes, fibroblasts, tumor cells, immune cells, and other components create the tumor microenvironment (TME), which causes tumor homeostasis. Exosomes’ composition can serve as prognostic indicators or the foundation for a system of grading the progression of cancer. Angiogenesis, oncogenesis, metastasis, tumor growth, and treatment resistance are all impacted by exosomes that are released by tumor cells (Figure 1) (Li et al., 2020; Gurung et al., 2021).

Nine million cancer-related deaths and 18 million new cases are recorded each year. Of these, more than 400,000 cases—or roughly 90% of them—are connected to childhood cancer and occur in developing nations. While this percentage is over 80% in European countries, survival rates in African nations are as low as 20% (Galanello et al., 2003). Chemotherapy, surgery, targeted therapy, and radiotherapy are common cancer therapies (Debela et al., 2021; Moha et al., 2021). The most efficient forms of cancer treatment include radiotherapy and chemotherapy, but they can have side effects like long-term issues and medication resistance (Debela et al., 2021; Keyvani et al., 2022). By influencing the immune system to reactivate the anti-cancer immune response, cancer immunotherapy can control and remove tumors (Ghasemi et al., 2020; Ghasemi et al., 2021; Hasan-Abad et al., 2022; Chiang et al., 2023). The Food and Drug Administration (FDA) of the United States has given its approval. There are many immunotherapy medications available for use in therapeutic settings, including inhibitors of the protein cytotoxic T-lymphocyte-associated protein 4 (CTLA-4) and programmed cell death 1 (PD-1) ligand 1 (Chiang et al., 2023). Exosomes have been found to have the dual properties of inhibiting and promoting cancer in recent investigations on the various functions they play in the development of the disease. Exosomes, also known as cell-derived nanovesicles, may be used in cancer immunotherapy due to their molecular transfer and immunogenicity properties (Xu et al., 2020).

Exosomes’ ability to convey information and their high loading efficiency is being increasingly used in research that aims to deliver functional RNAs or medicine. Moreover, by altering their surface indicators, exosomes can become capable of targeting tumors (Li et al., 2020). Consequently, exosome-based therapy is viewed as a viable approach to treating cancer. We concentrated on the exosome-based therapeutic approach in the current review to treat hematological malignancies.

## Exosome isolation methods

Exosome separation is essential for comprehending their mechanisms. Ultrafiltration, centrifugation, Immunocapture, size exclusion chromatography (SEC), microfluidics-based technologies, and affinity capture on antibody-coupled beads are examples of frequently used isolation techniques (Yang et al., 2020). The principles and characteristics of each method are explained below.

1. Ultracentrifugation: ultracentrifugation is a widely used method for exosome isolation. It involves sequential centrifugation steps to pellet and purify exosomes based on their size and density (Coughlan et al., 2020). The principle is to centrifuge the sample at high speeds to larger sediment particles, followed by ultracentrifugation to pellet exosomes. Advantages include relatively simple and cost-effective implementation. However, it can be time-consuming, requires specialized equipment, and may lead to co-isolation of other extracellular vesicles or contaminants (Taylor and Shah, 2015).
2. Density gradient centrifugation: this method separates exosomes based on their buoyant density (Yang et al., 2020). The sample is layered onto a density gradient medium and centrifuged, resulting in the formation of distinct bands corresponding to different vesicle populations. Exosomes can be collected from the appropriate density fraction. The principle is that exosomes with similar densities will migrate to specific regions in the gradient. Advantages include improved purity compared to ultracentrifugation. However, it requires longer processing time, gradient optimization, and can still result in the co-isolation of other vesicles (Li et al., 2017).
3. Size exclusion chromatography (SEC): SEC separates exosomes based on their size. The sample is loaded onto a column with porous beads, and smaller particles, including exosomes, elute later than larger particles. The principle is that exosomes can be separated from contaminants by their size exclusion. Advantages include simplicity, reproducibility, and minimal damage to exosomes. However, it may not provide high purity and can result in the loss of small-sized exosomes (Zhang and Lyden, 2019).
4. Immunocapture: this technique utilizes specific antibodies or affinity ligands immobilized on beads or surfaces to capture exosomes expressing specific surface markers. The principle is the selective binding of exosomes to the capture agent, allowing their isolation. Advantages include targeted isolation of specific exosomes and potential enrichment of subpopulations of interest. However, it relies on the availability of suitable antibodies, may result in co-isolation of non-exosomal material, and can have limited yield (Yang et al., 2022).
5. Microfluidics-based techniques: microfluidics platforms, such as microfluidic chips or nanostructures, enable precise manipulation and isolation of exosomes based on their size, surface markers, or other physical properties. The principle involves the controlled flow of samples through microchannels or nanostructures, allowing selective trapping or sorting of exosomes. Advantages include high specificity, minimal sample volume requirement, and potential for integration with downstream analysis. However, these techniques can be expensive, require specialized equipment, and have limited throughput (Lin et al., 2021).

The choice of isolation technique in clinical settings depends on various factors, including the type of bio fluid, required purity, sample volume, and downstream applications. Different techniques may be employed depending on the specific research objectives and available resources. It is worth noting that the field of exosome isolation is rapidly evolving, and new techniques are continually being developed and evaluated for their applicability in clinical settings. The most effective method for isolating exosomes is a centrifugation-based test. One of the greatest techniques for separating exosomes based on size, in addition to centrifugation, is ultrafiltration, which is now utilized in conjunction with ultracentrifuges (Yang et al., 2020). It has been demonstrated that exosomes can be found in the serum, urine, plasma, cerebrospinal fluid, and lymph of both healthy people and cancer patients (Mimeault and Batra, 2014).

## Role of exosomes in the development of cancer

The maintenance of physiological homeostasis and the development of disease symptoms depend on cell-to-cell communication. By using paracrine and autocrine signaling, exosome-associated proteins, DNAs, RNAs, miRNAs, and metabolites can change the fate of recipient cells. Exosomes secreted by tumors have been thoroughly investigated in a variety of cancer types, including melanoma, breast cancer, renal cancer, and hematological cancer. For instance, TGF-1, which is linked to the TGF-1 receptor on the surface of the leukemia cells, is present in exosomes released by chronic myeloid leukemia cells. This causes the exosomes to activate the AKT, ERK, and anti-apoptotic pathways in the producer cells, which in turn causes the tumor to proliferate (Raimondo et al., 2015). Other reports on glioma cancer cells showed the paracrine mechanism of tumor-released exosomes in the development and metastasis of cancer. They found that glioma cells can transfer extracellular vesicles (EVs) with the EGFRvIII (oncogenic receptor) to adjacent glioma cells that lack this receptor, therefore leading to activation of the AKT pathway in neighboring glioma cells. Therefore, it leads to the activation of the AKT pathway in the adjacent glioma cells and causes anchor-independent growth of these cells (Al-Nedawi et al., 2008; Hasan-Abad et al., 2019).

**FIGURE 2:**
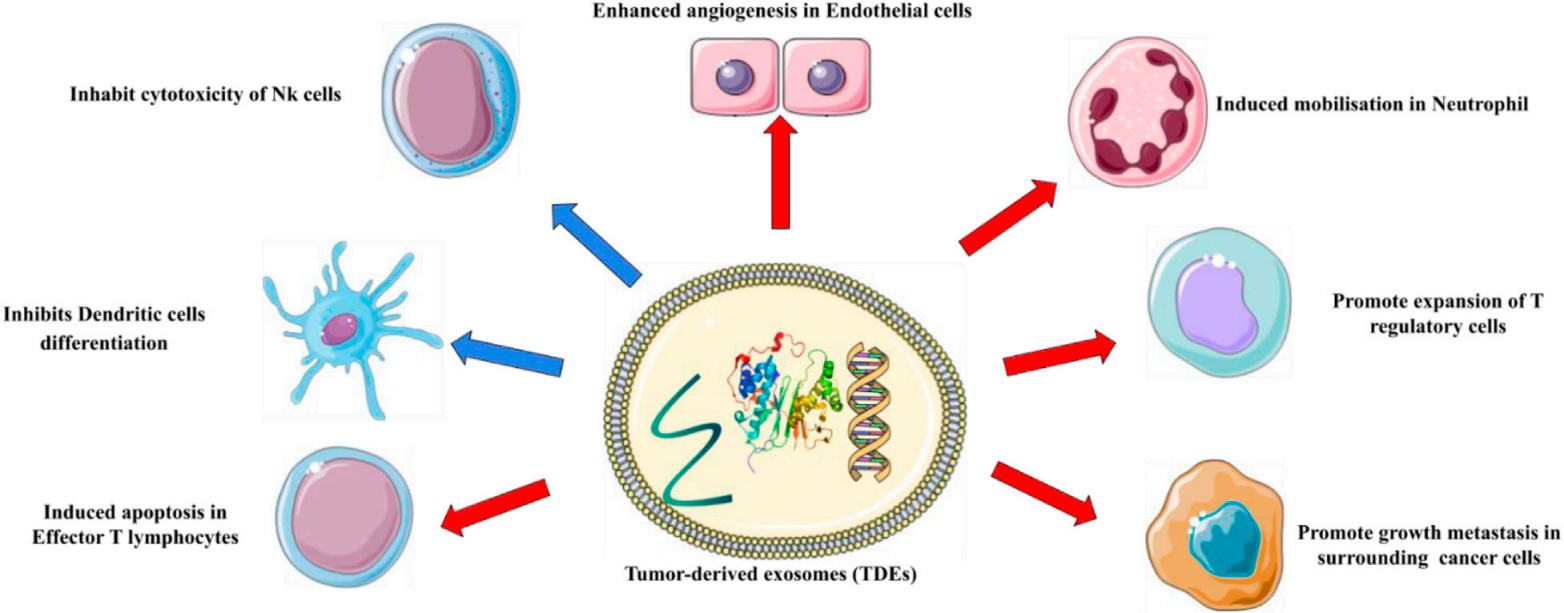
The roles of exosomes in cancer. Exosomes have been found to play a pivotal role in tumor initiation, growth, progression, and metastasis.

So far, researchers have discovered that in pediatric and adult patients with acute myeloid leukemia (AML), the level of dsDNA in EVs in plasma decreases after treatment but increases after relapse (Bernardi et al., 2021). It was recently shown that cancer stem cell exosomes transported miR-19b-3p to renal carcinoma cells, which then triggered epithelial-mesenchymal transition (EMT) to drive tumor metastasis (Wang L. et al., 2019). p120-catenin in exosomes released from hepatocellular carcinoma (HCC) cells, on the other hand, inhibited the expansion, metastasis, and proliferation of HCC CSCs (Cheng et al., 2019). Table 1 summarizes examples of exosomes involved in the advancement of hematological malignancies. These findings may aid in the understanding of the role of exosomes in the development of cancer and hematological malignancies, leading to improved therapeutic methods (Figure 2).

**TABLE 1:** The role of exosome cargos in the development of tumors.

Hematological malignancies	Exosome cargos	Action mechanisms	References
Adult T- cell leukemia (ATL)	Tax, AKT, Rb, cFLIP, NF-kB	Enhance cell survival in murine and human T-cell cell lines	Hong et al. (2014)
Chronic Myeloid Leukemia (CML)	miR-92a	Stimulated the development of vascular tubular structures	Ohyashiki et al. (2016)
	miR-365	Suppressed the production of Caspase3 and tumor cell apoptosis	Wang et al. (2014)
	hTERT mRNA	Enhanced the transformation of fibroblasts into CAF which aids leukemia cell survival	Webber et al. (2010)
	BCR-ABL1 mRNA	Induced BM-MSC to acquire a pro-tumor phenotype	Gross et al. (2012)
	Amphiregulin	Stromal cells’ EGFR signaling is activated, and their expression of IL-8 is enhanced	Kumar et al. (2018), Rezaie et al. (2021)
	MAC	Eliminated the MAC, which kills cancer, from malignant cells	Haderk et al. (2017)
	TGF-b	Enhanced the survival and growth of malignant cells	Gross et al. (2012)
Acute lymphoblastic leukemia (ALL)	Galectin-3, NF-kB	Promote ALL drug resistance	Hong et al. (2014)
Acute myeloid leukemia (AML)	Uncertain	Inhibited the activity of CD8^+^ T cells by downregulating the expression of JAK3 (Janus kinase 3) and CD3z in activated T cells	Reiners et al. (2013)
		Bcl-2, an anti-apoptotic protein, was expressed more frequently	Roccaro et al. (2013)
	FasL, PD-L1, TGF-b	Anti-tumor immune cells were suppressed	Wieckowski et al. (2009), Whiteside (2016), Fu et al. (2017)
	miR-20a miR-196	Ejected certain RNAs from cells that were not good for tumor survival	Chapuy et al. (2008)
	ABCA3	Anti-tumor medicines that are entrapped in membrane structures	Pilzer and Fishelson (2005)
	IGF-IR mRNA MMP9 mRNA	Increased stromal cell division and encouraged stromal cell secretion of growth factors	Rezaie et al. (2022)
	TGF-β1 MICA, MICB, ULBP1, ULBP2, BAG6	Reduce the ability of NK cells to kill leukemic cells	Cheng et al. (2013), Jaworski et al. (2014)
	IL-10, TGF-b	supported the transformation of CD4^+^CD25^−^ T cells into CD4^+^CD25+Foxp3+ T cells, or Treg cells	Reiners et al. (2013)
lymphoma	LMP1	increased proliferation and a malignant character in primary B-cell	Corrado et al. (2016)
	CK2	C9 was phosphorylated to safeguard malignant cells	Wang et al. (2016)
	MICA/B	NK cell cytotoxicity was decreased	Hong et al. (2017)
	Doxorubicin, pixantrone	cells were able to remove the chemotherapeutic medicines	Gong et al. (2013)
	CD20	lowered the bioavailability of rituximab when coupled with it	Torreggiani et al. (2016)
Multiple myeloma (MM)	miR-135b	reduced FIH-1, increased HIF-1 bioactivity, and encouraged angiogenesis	Naoyo et al. (2006)
	IL-6, CCL2, Fibronectin	Increased tumor cell survival, expansion, and migration	Hassanpour et al. (2020)
	Heparinase	encouraging macrophages to release TNF-a	Corrado et al. (2014)
	miR-146a, miR-16–5p, miR-15a-5p, miR-20a-50, miR-17–5p, bFGF	Transmits into MSCs and induces secretion of tumor-promoting cytokines Drug resistance Angiogenesis increase	Sezer et al. (2001), De Veirman et al. (2016), Zhang et al. (2016)
	Uncertain	Bcl-2 expression was elevated as a result of pro-survival signaling pathways such as JNK, p38, p53, and Akt being activated	Raimondo et al. (2015)
		STAT3 was controlled, MDSCs grew, and its immunosuppressive action was increased	Whiteside (2013)
Burkitt	LMP1	increased proliferation and a malignant character in primary B-cell	Corrado et al. (2016)
leukemia	MICA/B	NK cell cytotoxicity was decreased	Hong et al. (2017)
Chronic lymphocytic leukemia (CLL)	Y RNA hY4	induced an immunosuppressive phenotype in monocytes	Valenti et al. (2006)
	miR-21, miR-146a, miR-150, miR-155, S100-A9	Enhanced MSC proliferation, EC angiogenic activity, CLL cell survival and proliferation Drug resistance Activates the NF-kB pathway and leads to leukemia progression	Paggetti et al. (2015), Yeh et al. (2015), Prieto et al. (2017)
	Uncertain	enhanced angiogenesis and endothelial cell migration; increased stromal cell growth and inflammatory cytokines release	Umezu et al. (2014)

## Exosome-based tumor suppression strategies

Exosomes play important roles in intercellular contact in a variety of clinical disorders, including cancer, diabetes, and Alzheimer’s disease. Exosome cargoes produced from tumors are linked to cancer development. As a result, novel and safe cancer treatment techniques such as regulation of exosome production and secretion, exosome-mediated cell communication and employing exosomes as carriers, and removal of active exosomal cargos have been proposed. Furthermore, exosomes can be used as a diagnostic tool (Tai et al., 2018). A study found that modulating intracellular Ca2+ levels with dimethyl amiloride (DMA), an inhibitor of Ca2+/Na + exchange, reduced exosome secretion in mice with EL4 lymphoma (Savina et al., 2003). Koch et al. did another trial to inhibit exosome realization. They found that preventing exosomal drug resistance with indomethacin improved the effect of anthracene-dione pixantrone and anthracycline doxorubicin in DLBCL cell lines (Koch et al., 2016).

## The use of exosomes as carriers in the treatment of hematological malignancies

Exosomes can transport therapeutic molecules to cancer cells via acquired or innate ligands produced on their surfaces, such as proteins, nucleic acids, and other medicines. Recently, anticancer medicines doxorubicin and paclitaxel were successfully loaded into exosomes (Tian et al., 2014). Exosome drug delivery enhanced cancer targeting via an external magnetic field and reduced tumor growth, overcoming major challenges to the utilization of exosomes for cancer treatment. Exosomes containing the TRAIL (TNF-related apoptosis-inducing ligand) protein were found to increase apoptosis and prevent tumor growth in myeloid leukemia both *in vitro* (KMS11 multiple myeloma, INT12 melanoma, and B-cell lymphoma) and *in vivo* (mice) (Rivoltini et al., 2016a). Another study examined the anti-cancer effect of imatinib or BCR-ABL siRNA cargo with exosome on chronic myeloid leukemia (CML) *in vitro* (CML cell lines included: K562, imatinib K562R, and LAMA84 cells) and *in vivo* (mice). Exosomes target CML cells selectively with Lamp2b-IL3 and limit proliferation when loaded with BCR-ABL siRNA or imatinib by lowering the expression of the Bcr-Abl oncoprotein (Bellavia et al., 2017). Bellavia et al. employed HEK293 T cells that had been engineered to express the exosomal Lamp2b, which was attached to an Interleukin 3 (IL3) receptor. In contrast, despite normal cells, the IL3 receptor is overexpressed in CML blasts and acts as a therapeutic target in the cancer drug delivery system. They demonstrated that IL3L exosomes laden with the drugs BCR-ABL siRNA or Imatinib can target CML cells and limit cancer cell proliferation *in vivo* and *in vitro* (Bellavia et al., 2017).

## Exosomes as biomarkers in hematological malignancies

Exosomes can be extracted from a variety of bodily fluids, including urine, plasma, serum, saliva, breast milk, malignant ascites fluid, and amniotic fluid (Keller et al., 2011). Furthermore, biologically active EVS extracted from plasma could be employed as novel biological indicators. In the case of cancer, a suitable biomarker should be able to detect possibly metastatic tumors during the primary stage, while the cancer can still be treated (Testa et al., 2021). EVs produced by cancer cells contain tumor-specific components that can shield their contents from proteases and nucleases while also increasing biomarker half-life. Recently, it was shown that miR-155 can be used as a biomarker in patients with monoclonal B-cell lymphocytosis and B-cell leukemia (Ferrajoli et al., 2013). Several investigations have proven the use of exosomal bioactive compounds as prognostic and diagnostic markers in other forms of cancer. The RNA biomarker AR-V7 RNA expression level, for example, increases during prostate cancer and can be detected using the PCR method. The level of the protein biomarker MIF in the plasma increases in pancreatic cancer, which may be identified using the ELISA method. Other biomarkers, such as EGFR vIII mRNA in glioblastoma (in plasma), integrin in breast cancer (in plasma), and ZFAS1 long noncoding RNA in gastric cancer (in serum), are examples of how exosomes can be used to diagnose cancer (Tai et al., 2018).

Except for CML (Philadelphia chromosome as a biomarker), the lack of particular molecular markers capable of being exploited has had a significant impact on the early diagnosis and treatment of these cancers. Several studies have found that mutations or aberrant expression of certain mRNAs or miRNAs are linked to hematological malignancies (Ferrajoli et al., 2013). Exosomal miR150, miR155, and miR1246 levels have been found to rise in AML patients (Hornick et al., 2015). The differential centrifugation method or the miRNeasy kit can be used to assess the serum or plasma level of certain miRNAs. In MM patients, lowering serum levels of exosomal miR20a-5p, miR16-5p, miR15a-5p, and miR16-5p has the potential to be used in the separation or construction of prognostic and diagnostic panels (Zhang et al., 2016).

## Cell-derived exosomes as therapeutic agents

### MSC-derived exosomes

In malignancies or diseases, MSC-exosomes can carry complicated payloads and affect body homeostasis (Lou et al., 2017). In the previous decade, MSC-exosomes have been used to treat a variety of hematological disorders including multiple myeloma (MM), myelodysplastic syndrome (MDS), lymphoma, GVHD, CLL, AML, and CML (Shen and Chen, 2021). They have a role in tumor growth by inducing matrix remodeling and angiogenesis in a pro-tumor niche, inhibiting apoptosis and the activity of anti-tumor immune cells such as CTL and NK cells, and delivering pro-survival cytokines (Shen and Chen, 2021). For example, there is evidence that bone marrow MSCs promote the formation of MM by secreting interleukin-6 (IL-6) as a tumor cell growth factor (Lou et al., 2017; Shen and Chen, 2021). MSCs are pluripotent cells with very diverse characteristics that play a dual function in cancer formation. This topic explains why clinical trials examining the effect of MSCs on cancer have been equivocal and have not progressed beyond phase 1. Approximately 42 clinical trials were registered on www.clinicaltrials.gov until 2019, with a focus on the efficacy of MSCs on malignancies, with just 13 focusing on hematologic malignancy. Only one of the 13 clinical trials looked at the anticancer effects of MSCs. Other investigations were linked to the immunomodulatory function of MSCs after stem cell transplantation (Rappa et al., 2012; Lou et al., 2017; Lee et al., 2019). As a result, more research is needed to comprehend the anti-cancer applicability of MSCs.

MSC anticancer mechanisms are still unknown. However, some of them are desirable for use in the prevention of hematological malignancies. Exosome-derived cancer cells are being studied for ways to silence or eliminate them (Lee et al., 2019). Exosome removal from the circulation (via plasmapheresis), prevention of exosome production (via DMA), exosome suppression (via silencing of targeting ESCRT proteins or GTP-ase Rab27A), prevention of endolysosomal contents functions (via Proton pump inhibitors (PPI), and prevention of exosome uptake or fusion by target cells (via blocking phosphatidyl serine with diannexin) are all under investigation strategies for (Vader et al., 2014). However, because of the essential and dual functions of exosomes, more research is required to develop an authorized safe cancer therapeutic technique.

According to ongoing research, MSC exosomes can infiltrate tumor locations due to an increased retention (EPR) impact and permeability, making them suitable delivery vehicles for suppressing cell proliferation and vascularization (Naseri et al., 2018). For example, combining modified MSC with medication-loaded nanoparticles containing the anticancer agent paclitaxel results in a significant reduction of tumor growth and survival *in vivo* (Naseri et al., 2018). Furthermore, the findings of this study revealed that using nano-engineered MSCs as a drug vehicle considerably increased the anticancer efficiency of traditional chemotherapy medicines (Layek et al., 2018). A study found that combining MSCs expressing exosomal siGRP78 with sorafenib could reduce proliferation, invasion, and reverse drug resistance in hepatocellular carcinoma cells (Li H. et al., 2018). There are now multiple clinical trials using exosome-based treatments for solid tumors, including lung cancer (23 studies), breast cancer (12 studies), renal cancer (three studies), and ovarian cancer (three studies) (www.clinicaltrials.gov website, keyword exosome). However, just two studies on the use of exosomes for non-Hodgkin B-cell lymphoma therapy were found in the field of hematologic malignancies (Fernandes et al., 2019). Several studies have found that MSCs inhibit both apoptosis and proliferation, enhance cell proliferation, and aid in tumor vasculature (Layek et al., 2018; Lee et al., 2019; Shen and Chen, 2021). Furthermore, because of the immunomodulatory properties of MSCs, which increase the rate of recurrence and metastasis, leading to chemo-resistance, and tending to shield tumor cells from drug-induced death, MSCs are not suitable for therapeutic usage (Chen et al., 2018; Lee et al., 2019).

### Dendritic cell-derived exosomes

DCs have emerged as the most potent antigen-presenting cells (APCs) for cancer vaccinations in recent years. Several studies and phase I or II clinical trials have been done to monitor the anticancer effects of DCs (Pyzer et al., 2014). WT1 (Wilm’s tumor 1 protein) mRNA-electroporated DC vaccination was created by Van Tendeloo et al. They vaccinated ten AML patients in a phase I/II experiment. Vaccinated patients had an increase in WT1-specific T cells as well as an increase in WT1-specific IFN-producing CD8^+^ T cells (Van Tendeloo et al., 2010).

Although the DC-based vaccine provided benefits such as activation of T-helper cells, killer T cells, and B-cell, as well as induction of cancer-specific T-cell response, there were numerous disadvantages. Tumor cells, for example, may produce immunosuppressive soluble cytokines that transform DCs into tolerogenic DCs, resulting in T-reg cell activation (Palucka and Banchereau, 2012; Moradi Hasan-Abad et al., 2022). The use of exosomes produced from DCs (Dex) has been proposed as a remedy to the technical hurdles associated with DC-based immunotherapy (Andre et al., 2004). For example, in comparison to DCs, Dex has exceptional stability at −80°C for 6 months due to its high lipid makeup. Furthermore, Dex expresses adequate ligands for NK cell receptors and is resistant to immunosuppressive compounds and TME immunoregulation (Pitt et al., 2016).

Recently, a novel CML-RAE-1-Dex vaccination with RAE-1 expression was developed. RAE-1 is a key ligand of NK group 2 D (NKG2D), which is required for T- and NK-cell responses. Their discovery demonstrated an anticancer effect both *in vitro* and *in vivo*. Their Dex vaccination boosted the effector functions and proliferation of NK cells, CD8^+^ T cells, and CD4^+^ T cells, resulting in a strong anti-CML impact *in vitro*. Furthermore, in CML animal models, immunotherapy with CML-RAE-1-Dex reduced leukemogenesis and established long-lasting immunological memory (Du et al., 2022). Immune responses were similar in imatinib-resistant CML cells with the T315I mutation (Du et al., 2022).

Yao et al. employed an LEXs-pulsed DCs vaccination against leukemia in 2014. Exosomes produced from K562 leukemia cells (LEXK562) include immunologically related molecules (hsp70) as well as membrane-bound vesicles containing adhesion molecules (intercellular adhesion molecule-1). In comparison to LEXs and non-pulsed DCs, LEXs-pulsed DCs elicited a stronger anti-leukemic cytotoxic T-cell response (Yao et al., 2014). Dex-based vaccination technology is one of the areas of interest for researchers as the number of preclinical and clinical investigations on hematological malignancies continues to rise.

### Natural killer cell-derived exosomes

Patients with AML exhibit a low NK-cell frequency and significantly lower NK cell activity in the peripheral blood, as well as lower expression of NCR, NKp46, NKp44, NKp30, and C-type lectin receptors, NKG2C and NKG2D (Szczepanski et al., 2011). In AML, NK cell functions (intracellular signaling, cytokine generation, and cytotoxicity) are reduced; NK cell dysfunction is probably associated with the disease’s initial expansion, progression, or recurrence (Shimasaki et al., 2020).

Exosomes containing essential cytotoxic chemicals such as LFA-1, IFN-, DNAM1, and PD-1 are produced in large amounts by stimulated NK cells (Shimasaki et al., 2020).

In neuroblastoma tumor-bearing animals, NK-92 cell-exosomes used as a drug delivery vehicle had good targeting capacity, with robust fluorescence observed 6 h after injection; in a subcutaneous tumor-bearing mouse model, exosomes were found in tumor cells 20 min after injection (Wang G. et al., 2019). Furthermore, exosomes released by NK cells with IL-15 had a greater targeting ability, were faster acting, and persisted in the tumor site and blood circulation for a longer period, with a half-life of up to 24 h (Zhu et al., 2019).

Another ability of NK cells to fight cancer is the production of exosomes or MVs containing cytotoxic proteins and miRNA. Granzyme A and B, Perforin, and granulysin have antitumor effects on NK cells by activating apoptotic pathways via caspase-dependent and -independent apoptotic pathways, single-stranded DNA damage, mitochondrial disruption resulting in cytochrome c release and activation of the apoptotic cascade, induces ER stress-mediated apoptosis, and damages the cell membrane. The anti-cancer action of miR-186 released by NK cells was demonstrated by reducing neuroblastoma cell proliferation and causing apoptosis (Wu et al., 2021).

Furthermore, when NK cells are pre-exposed to neuroblastoma cells, they release exosomes with greater expression of different activating receptors such as NKp46, NKp44, NKp30, and NKG2D, as well as enhanced cytotoxicity when compared to untreated NK cells. This work shows that, in addition to their cytotoxic role, NK cells can be positively impacted by neuroblastoma cells, resulting in efficient cytotoxicity against neuroblastoma tumors (Shoae-Hassani et al., 2017). These findings provide hope for NK cell-based cancer therapy. More research is needed, however, to extrapolate these findings to other forms of cancer and hematological malignancies. Boyiadzis et al. investigated the efficacy of NK cell exosomes on AML cell lines (Kasumi, MLL-1), K562 targets, and primary leukemia blasts. They discovered that NK cell-derived exosomes containing killer-cell immunoglobulin-like receptors, TGF, perforin, NKG2D, granzyme B, and PD-1 increase blast cell lysis and have anti-leukemia action against K562 targets and AML cell lines (Boyiadzis et al., 2018).

Di Pace and others discovered that a novel marker called DNAX Accessory Molecule-1 (DNAM1) is involved in the exosome-mediated cytotoxicity of NK cells in a children B acute lymphoblastic leukemia cell line. Furthermore, they report that the cytotoxic action of NK cell-derived exosomes was dose-dependent, with the percentage of killed cells increasing as exosome concentrations increased, reaching a maximum of 50. This feature suggests that they could be used in immunotherapy (Di Pace et al., 2020).

Despite their vital role in cancer defenses, NK cells’ application in cancer treatment is limited due to their diminished ability to reach tumor sites and the inhibitory effects of TME on their function or diminution of anti-cancer action within a few hours (Labani-Motlagh et al., 2016; Li Q. et al., 2018; Di Pace et al., 2020).

Future studies should focus on increasing the site specificity and durability of the anti-cancer response mediated by NK cell-generated exosomes via genetic engineering to better exploit these potent cells in cancer therapy. Exosome-mediated treatment approaches for hematological Cancers are shown in Table 2.

**TABLE 2:** Exosome-mediated treatment approaches for the treatment of hematological cancers.

Hematological malignancies	Exosomes	Cancer cell line or target	Finding	References
Acute myeloid leukemia (AML)	hMS-bone marrow (BM)Arsenic trioxide and derived exosomes	NB4 cell line	more significant amounts of apoptosis markers	Abbaszade Dibavar et al. (2018)
	BM-MSC-exo treatment	NB4 cell line	S100A4 deficiency reduced leukemia cell proliferation and dispersion	Lyu et al. (2021)
	Exosomes from stimulated NK cells contain key cytotoxic chemicals such as IFN-g, lymphocyte function-associated antigen (LFA-1), DNAX accessory molecule-1 (DNAM1), and programmed cell death protein (PD-1)	NK cells	Increase the immunological response to AML	Di Pace et al. (2020)
	PEGylated liposomal doxorubicin (PLD)/GW4896	U937 cells	make U937 cells more susceptible to the cytotoxic effects of PLD	Hekmatirad et al. (2021)
	Blocking exosomes	HS-5 BMSCs	etoposide-induced apoptosis was boosted	Chen et al. (2019)
Acute Lymphoblastic Leukemia (ALL)	exosome-derived miRNA-181a		ExomiR-181a inhibition reduced the expression of genes that promote cell proliferation, such as BCL2, PCNA, MCL-1, and KI-67. Furthermore, blocking pro-apoptotic genes like BAX or BAD can limit exosome-induced cell proliferation	Haque and Vaiselbuh (2020)
	Exosomes derived from leukemia (LEXTGF-1si)	LEX cell lines	TGF-1 suppression by shRNA, decrease of leukemia cell proliferation, and increased survival in an animal model	Huang et al. (2018)
	Chimeric antigen receptor-exosome Cd19	CD19-positive B-cell neoplasms	Increased cellular toxicity and programmed cell death	Haque and Vaiselbuh (2021)
Chronic myeloid leukemia (CML)	Imatinib-armed IL3-exosomes	CML blasts	*In vitro* and *in vivo* inhibition of leukemia cell growth	Bellavia et al. (2017)
	venetoclax-armed immunoliposome (IL-VX)	CML cell line	induce Apoptosis	Houshmand et al. (2021)
	exosomal elimination of miR-21 in curcumin plasma	CML xenograft in SCID mice	Exosome miRNA-21 may contribute to curcumin’s antitumoral effects in CML	Taverna et al. (2015)
	LAMA84-originated exosomes	leukemic cells	Exosomes increased cell proliferation and produced an antiapoptotic phenotype	Raimondo et al. (2015)
	BM MSC-Exo	CML cells	Through miRNA-15a, inhibit CML cell proliferation *in vitro* and trigger apoptosis	Zhang et al. (2020b)
	Mesenchymal stromal cell exosomes (hUC-MSC-Exo)	K562 951 cells	Suppression of induced cell viability and apoptosis	Liu et al. (2018)
Lymphomas	Curcumin is contained within exosomes derived from lymphoma cells	CD11b+/Gr–1+ cells	Increases programmed cell apoptosis and has anti-inflammatory properties	Sun et al. (2010)
	TRAIL + exosomes	SUDHL4-bearing mice	Reduction In Lymphoma Multiplication	Rivoltini et al. (2016b)
	Exosome-like nanoparticles carrying siRNA	L820 lymphoma cells	In l820 lymphoma cells, inhibiting c-Myc effectively activated poly (ADP-ribose)polymerase-dependent apoptotic systems	Lunavat et al. (2016)
	Gp350c exosomes	CD21 on B-cell	Powerful immunogenic effect, stimulating the proliferation of tumor-associated and EBV-specific T cells	Ruiss et al. (2011)
Multiple Myeloma	Exosomes were produced by cells that had been exposed to antibodies	MM cells (BF01)	NK cells exhibited a decrease in the expression of genes involved in the cell cycle and an increase in the expression of genes associated with the stimulation of the immune response	Malavasi et al. (2021)
	HSP70-modified exosomes derived from MM cells	Dcs	Triggered effective CD4+/Th1, CD8+/CTL, and NK-mediated anti-MM immune response	Xie et al. (2010)
	Exosomal circrna	H9C2 cells	Exo-circrnas could develop into a novel diagnostic marker for myocardial injury	Sun et al. (2021)

## Challenges and limitations of the therapeutic use of exosomes

The unique properties of exosomes, such as stability, biocompatibility, and ability to transport bioactive molecules, make them promising candidates for therapeutic interventions. The therapeutic use of exosomes holds great promise, but several challenges and limitations need to be addressed for successful clinical translation. Overcoming these challenges requires interdisciplinary collaboration, innovative technologies, and a deep understanding of the complex biology of exosomes. The most important challenges and limitations of the therapeutic use of exosomes are explained below.

1. Isolation and purification: one of the key challenges is the development of standardized and reproducible methods for isolating and purifying exosomes (Ayala-Mar et al., 2019). Current isolation techniques, such as ultracentrifugation and immunocapture, have limitations in terms of scalability, purity, and yield. There is a need for robust and efficient isolation methods that can provide highly purified exosome populations suitable for clinical applications (Ayala-Mar et al., 2019; Liangsupree et al., 2021).
2. Heterogeneity: exosomes derived from different cell types or even from the same cell type can exhibit significant heterogeneity in terms of cargo composition and functional properties (O’Brien et al., 2020). Understanding and controlling this heterogeneity is crucial for ensuring consistent therapeutic outcomes. Characterization techniques and quality control measures need to be improved to assess the heterogeneity of exosomes and establish standardized criteria for their therapeutic use (Willis et al., 2017).
3. Scalability and manufacturing: the clinical translation of exosome-based therapies requires scalable and cost-effective manufacturing processes (Amondarain et al., 2023). Currently, the production of exosomes in large quantities remains a challenge. Developing scalable manufacturing platforms, including bioreactors and microfluidic systems, is necessary to meet the demands of clinical applications (Colao et al., 2018; Pan et al., 2023).
4. Targeted delivery: efficient and targeted delivery of exosomes to specific tissues or cells is essential for achieving desired therapeutic effects (Rehman et al., 2022). Overcoming biological barriers, such as clearance by the immune system and off-target effects, is critical for successful targeted delivery. Strategies like surface modification and engineering approaches need to be explored to enhance the specificity and efficiency of exosome delivery (Mitchell et al., 2021; Waheed et al., 2022).
5. Regulatory considerations: the therapeutic use of exosomes falls under regulatory frameworks that govern biological products and drug delivery systems (Bunggulawa et al., 2018). Understanding and complying with these regulations is crucial for the successful translation of exosome-based therapies from preclinical studies to clinical trials and commercialization (Willis et al., 2017).
6. Clinical translation and efficacy: although preclinical studies have shown promising results, the efficacy of exosome-based therapies in clinical settings is still being explored. The development of well-designed clinical trials and rigorous evaluation of therapeutic outcomes are necessary to determine the safety, efficacy, and long-term effects of exosome-based interventions.

## Future of the therapeutic use of exosomes

The advancements in exosome research have the potential to transform cancer treatment by providing novel therapeutic strategies. Further investigation in these research directions will not only expand our knowledge of exosome biology but also pave the way for personalized, targeted, and more effective cancer therapies, ultimately improving patient outcomes and quality of life. Several areas warrant further investigation to harness the full potential of exosomes and improve patient outcomes.

Cargo and Functional Analysis: Future research should focus on elucidating the specific cargo components carried by exosomes and their functional relevance in cancer treatment (Zhang et al., 2019). Identifying the key molecules, such as miRNAs, proteins, and lipids, involved in modulating tumor growth, metastasis, immune response, and drug resistance will enhance our understanding of exosome-mediated intercellular communication and facilitate the development of targeted therapies (Mashouri et al., 2019).

Engineering and Modification: Investigating exosome engineering strategies, such as surface modification and cargo loading techniques, can enhance their targeting ability and therapeutic efficacy (Salunkhe et al., 2020). Further exploration of bioengineering approaches, including genetic engineering and synthetic biology, can enable the customization of exosomes for specific cancer types and individual patient needs.

Delivery Optimization: Improving the efficiency and specificity of exosome delivery to tumor sites remains a critical area of investigation (Subhan and Torchilin, 2019). Developing novel delivery methods, such as exosome-based drug carriers or nanoscale delivery systems, can enhance the stability, biodistribution, and cellular uptake of exosomes, leading to improved therapeutic outcomes and reduced off-target effects (Subhan and Torchilin, 2020).

Biomarker Discovery: Identifying reliable exosomal biomarkers for cancer diagnosis, prognosis, and treatment response prediction is an important research direction (Li et al., 2022). Comprehensive profiling of exosomal molecular signatures in large patient cohorts, combined with advanced bioinformatics approaches, can aid in the identification of biomarkers that can guide personalized treatment decisions and improve patient stratification.

Clinical Translation: Conducting well-designed clinical trials to evaluate the safety and efficacy of exosome-based therapies in cancer patients is crucial. Longitudinal studies assessing the long-term effects and monitoring potential adverse events associated with exosome treatments are needed. Collaboration between academia, industry, and regulatory bodies is essential to navigate the regulatory landscape and facilitate the translation of exosome research into clinical practice (Table 3).

**TABLE 3:** Some clinical trial on therapeutic use of exosomes in cancers, exosome type and outcomes.

Clinical trial ID	Cancer type	Exosome type	Results/Outcomes	Year
NCT03608631	Non-small cell lung cancer	Dendritic cell-derived exosomes	Ongoing trial, results not available yet	Ongoing
NCT03608631	Pancreatic cancer	Autologous dendritic cell-derived	Ongoing trial, results not available yet	Ongoing
NCT03437759	Acute myeloid leukemia	Mesenchymal stem cell-derived exosomes	Ongoing trial, results not available yet	Ongoing
NCT03437759	Chronic lymphocytic leukemia	Dendritic cell-derived exosomes	Ongoing trial, results not available yet	Ongoing
NCT04386049	Multiple myeloma	Mesenchymal stromal cell-derived	Ongoing trial, results not available yet	Ongoing
NCT03608631	Metastatic colorectal cancer	Mesenchymal stromal cell-derived	Ongoing trial, results not available yet	Ongoing
NCT03213314	Melanoma	Dendritic cell-derived exosomes	Ongoing trial, results not available yet	Ongoing
NCT03608631	Hepatocellular carcinoma	Autologous dendritic cell-derived	Ongoing trial, results not available yet	Ongoing
NCT03608631	Breast cancer	Dendritic cell-derived exosomes	Ongoing trial, results not available yet	Ongoing
NCT04386049	Hodgkin lymphoma	Dendritic cell-derived exosomes	Ongoing trial, results not available yet	Ongoing
NCT03608631	Non-Hodgkin lymphoma	Mesenchymal stromal cell-derived	Ongoing trial, results not available yet	Ongoing
NCT04421136	Polycythemia vera	Mesenchymal stromal cell-derived	Ongoing trial, results not available yet	Ongoing
NCT03608631	Ovarian cancer	Mesenchymal stromal cell-derived	Ongoing trial, results not available yet	Ongoing
NCT03608631	Acute lymphoblastic leukemia	Dendritic cell-derived exosomes	Ongoing trial, results not available yet	Ongoing
NCT03608631	Prostate cancer	Autologous dendritic cell-derived	Ongoing trial, results not available yet	Ongoing
NCT03873734	Myeloproliferative neoplasms	Mesenchymal stromal cell-derived	Ongoing trial, results not available yet	Ongoing
NCT03213314	Gastric cancer	Mesenchymal stromal cell-derived	Ongoing trial, results not available yet	Ongoing
NCT03608631	Myelodysplastic syndromes	Mesenchymal stromal cell-derived	Ongoing trial, results not available yet	Ongoing
NCT04276987	Head and neck cancer	Dendritic cell-derived exosomes	Ongoing trial, results not available yet	Ongoing
NCT03873734	Chronic myeloid leukemia	Dendritic cell-derived exosomes	Ongoing trial, results not available yet	Ongoing
NCT04045405	Bladder cancer	Autologous dendritic cell-derived	Ongoing trial, results not available yet	Ongoing
NCT04421136	Acute promyelocytic leukemia	Dendritic cell-derived exosomes	Ongoing trial, results not available yet	Ongoing
NCT04276987	Esophageal cancer	Mesenchymal stromal cell-derived	Ongoing trial, results not available yet	Ongoing
NCT04045405	Leukemia	Dendritic cell-derived exosomes	Ongoing trial, results not available yet	Ongoing
NCT04276987	Brain cancer	Mesenchymal stromal cell-derived	Ongoing trial, results not available yet	Ongoing

## Conclusion

The advancements in exosome research have the potential to transform cancer treatment by providing novel therapeutic strategies. Further investigation in these research directions will not only expand our knowledge of exosome biology but also pave the way for personalized, targeted, and more effective cancer therapies, ultimately improving patient outcomes and quality of life.

Exosomes are a relatively young field of study. Exosomes have sparked great attention in the realm of cancer therapy due to their potential use as new low-toxicity inhibitors in immunotherapy and diagnostic biomarkers, as well as a safer and more efficient method of delivering anti-cancer medications. However, there are various obstacles to their optimal utilization. Methods for large-scale manufacturing, isolation, and storage, in particular, should be carefully tuned. Furthermore, the therapeutic potential and delivery efficiency of exosomes, the route of administration, and the immunologic influence of exosomes on cancer cell suppression must all be carefully examined. In the case of hematological malignancies, concentrating on the use of exosomes for drug delivery, developing protocols for the prevention of drug resistance mediated by exosomes (such as using monoclonal antibodies, aptamers, nanoparticles, engineered and modified exosomes, *etc.*), and developing methods for blocking exosomes associated with the progression or metastasis of hematological malignancies can all contribute to better cancer therapy management in the future.
